# Perceived barriers and facilitators of staff recruiting participants to a randomised controlled trial of a community rehabilitation intervention following hip fracture

**DOI:** 10.1186/s13063-024-08655-z

**Published:** 2024-12-18

**Authors:** Kathryn Harvey, Penelope Ralph, Llinos Haf Spencer, Kodchawan Doungsong, Val Morrison, Andrew Lemmey, Miriam Golding-Day, Susanna Dodd, Ben Hardwick, Shanaz Dorkenoo, Sophie Hennessey, Nefyn Williams

**Affiliations:** 1 Department of Primary Care and Mental Health, University of Liverpool, Liverpool, UK; 2 Faculty of Life Sciences and Education, University of South Wales, Newport, UK; 3 Centre for Health Economics and Medicines Evaluation, Bangor University, Gwynedd, UK; 4 School of Psychology and Sport Science, Bangor University, Gwynedd, UK; 5 Faculty of Medicine and Health Sciences, University of Nottingham, Nottingham, UK; 6 Department of Health Data Science, University of Liverpool, Liverpool, UK; 7 Faculty of Health and Life Sciences, University of Liverpool, Liverpool, UK; 8 Llandrindod, UK

**Keywords:** Qualitative, Community healthcare complex intervention, Rehabilitation, Elderly medicine, Recruitment

## Abstract

**Background:**

Randomised controlled trials (RCTs) often struggle with recruitment and many need extensions which leads to delayed implementation of effective interventions. Recruitment to complex intervention trials have similar difficulties. Alongside this, the COVID-19 pandemic had a major impact upon trial recruitment. Research has shown that many other recruitment issues can be anticipated, for example overestimating target population prevalence; however, a range of factors may play a role. The aim of this study is to investigate facilitators and barriers to recruitment from the perspective of the recruiter.

**Methods:**

Fracture in the Elderly Multidisciplinary Rehabilitation – phase III (FEMuR III) was a RCT of a complex intervention post-surgery for hip fracture in patients over 60 years old. A process evaluation was undertaken, and semi-structured interviews were conducted with seven recruiters between November 2022 and March 2023 to identify barriers and facilitators to recruitment. A thematic analysis was undertaken in NVIVO (Version 12) using a critical realist perspective.

**Results:**

The trial took place mostly during the COVID-19 pandemic, and the unique impact of this on reported barriers is considered. A key finding included recruiter reluctance to approach patients that they felt would not benefit from the trial due to other factors (e.g. comorbidities or complex living situations). A possible barrier to recruiting carers appeared to be that family members did not relate to the label of ‘carer’ and so did not take part. Facilitators included recruiters approaching patients with other clinical or research staff. This approach, which included tailored initial information on the trial, reduced participant stress by increasing patient familiarity with recruiting staff and allowing staff time to develop relationships with patients.

**Conclusion:**

This paper identifies barriers and facilitators of recruitment to FEMuR III with six broad themes for both barriers and facilitators identified in the qualitative data synthesis. The impact of the COVID-19 pandemic was the main, but not sole, barrier to recruitment. Key findings concern reluctance to approach some eligible patients, the label of ‘carer’, the involvement of clinical staff and patient preference for trial group. Strategies to identify and overcome recruitment problems are highlighted and should be implemented and evaluated in further RCTs of complex interventions.

**Trial registration:**

ISRCTN28376407. November 23, 2018.

**Supplementary Information:**

The online version contains supplementary material available at 10.1186/s13063-024-08655-z.

## Introduction

Poor recruitment of participants into randomised controlled trials (RCTs) across a range of conditions and contexts is a common problem [1, 2]. A review of 114 RCTs funded by two UK agencies found that under a third achieved their original recruitment target and over half required an extension [1]. Another review of RCTs funded by the Health Technology Assessment Programme found that, between 2004 and 2016, only 85 of 151 RCTs (56%) achieved their recruitment targets [2]. A systematic review of 172 discontinued RCTs found that reasons for discontinuation included overestimating the number of eligible patients (*n* = 71), bias against the effectiveness of interventions from recruiters (*n* = 34) or participants (*n* = 33) and increased workload for patients (*n* = 20) and recruiters (*n* = 14) [3].

There are many reasons patients choose to take part, or not to take part, in trials. An overview of 26 systematic reviews in 2020 identified many patient-reported factors involved in the decision to take part in research, including perceived personal benefits, trust in their doctor, perceptions of research, knowledge, fear of side effects from the intervention and health status [4]. In 2018, a systematic review of 27 studies found that patient preference for an experimental or control group could also influence their decision to take part as they did not want to risk non-allocation to their preference [5]. In relation to the healthcare professionals’ perspectives on recruitment, qualitative studies have found that having appropriate resources and good relationships between the research team and clinicians facilitated recruitment [6, 7]. Barriers included disconnection between the clinical and research teams, restrictive recruitment criteria and ethical considerations which prevented them from approaching eligible patients [6, 7].

Recruitment to RCTs of rehabilitation is often challenging. A systematic review looking at recruitment to stroke rehabilitation RCTs between 2005 and 2015 found that for 321 RCTs the median randomisation rate was 34% with one to two patients recruited per site per month [8]. Screening those in the community or chronically affected by stroke was more likely to lead to randomisation than screening those in hospital or acutely unwell with stroke. Suggested reasons for this included patients feeling more able to partake in a trial after the immediate physical and psychological effects of their illness. In this paper, we report on the recruitment difficulties of another RCT of rehabilitation following hip fracture called FEMuR III (Fracture in the Elderly Multidisciplinary Rehabilitation – phase III) [9].

### Background to the FEMuR III RCT

FEMuR III was a pragmatic RCT examining the effectiveness and cost-effectiveness of an enhanced rehabilitation programme following surgical repair of hip fracture, compared with usual care [9]. Participants were older adults (aged ≥ 60 years) with mental capacity, living independently, recruited on orthopaedic wards whilst recovering from surgical repair. Friends and family, who identified as their carer, were also recruited. Carers were defined as helping the patient for 4 or more days a week with activities of daily living or physical care [9]. The enhanced rehabilitation intervention was delivered in the community following hospital discharge.

Recruitment procedure varied by site. Some sites had research nurses who identified eligible patients before informing the recruiters whilst other sites relied on ward staff informing recruiters about eligible patients who recruiters could then approach. Where available, electronic health records were accessed by recruiters to help identify eligible patients, but access varied by staff member. The recruitment process involved multiple visits to allow patients time to read information or discuss the trial with their families. In some sites, the community FEMuR III intervention was provided by the same acute trust that had provided in-patient care, whilst at other sites the intervention was provided by a separate community trust or healthcare organisation. Recruitment was permitted in both hospital and community settings after discharge. Amendments during the trial facilitated recruitment by extending the length of the eligibility window from 4 weeks post-surgery to 6 weeks post-surgery from 3/2/2020. A shortened patient information and carer information leaflet was also introduced as a substantial amendment and received Research Ethics Committee (REC) approval (24/6/2020) and Health Research Authority (HRA) approval (19/6/2020).

**Fig. 1:**
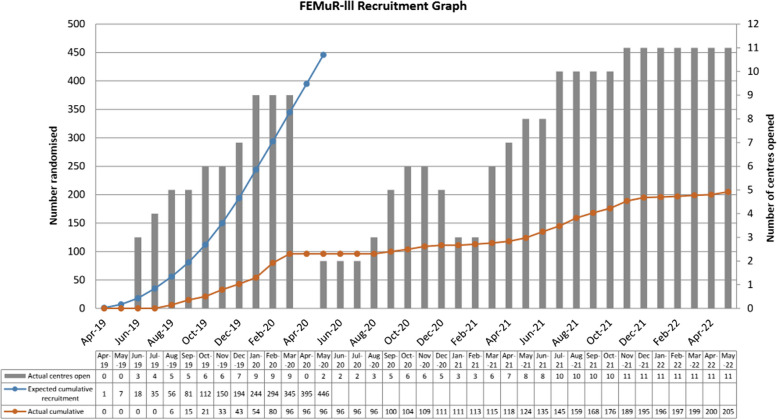
FEMuR III Recruitment Graph showing number of centres opened alongside number of participants randomised

Trial recruitment had been tested in an earlier feasibility study, which recruited 62 patient-participants and 31 carer-participants over 10 months from two acute hospital sites [10]. However, recruitment to the main RCT was severely affected by the COVID-19 (Coronavirus disease 2019) pandemic which began in 2020. Resources were focused on COVID-related research and non-essential access to clinical areas was blocked. Recruitment paused on 19/3/2020 and was on hold until 26/6/2020. Sites varied in the timing of re-opening for recruitment and closed to recruitment in response to local outbreaks. Recruitment struggled to pick up when it resumed. Recruitment was halted after 205 patient participants (46% of target sample size) and 15 carer participants had been recruited, which fell short of the target sample size of 446 (recruitment trajectory shown in Fig. 1). Recruitment was halted by the funder due to the poor recruitment rate as a consequence of the lockdown restrictions during the COVID-19 pandemic. Although COVID was a main barrier to recruitment, 96/205 participants were recruited prior to the pandemic so there were likely other factors that made recruitment challenging.

The aim of this study was to explore the facilitators and barriers to recruitment to the FEMuR III RCT from the perspective of the staff recruiting participants.

## Methods

All staff involved in recruitment to the FEMuR III RCT were approached by email from P.R. (PhD) who was employed as a research assistant for the trial. The decision to interview the recruiters was a later amendment to the protocol (approved October 2022). Purposive sampling was used. They were asked if they would take part in interviews related to their role in the study. Potential participants were likely aware of P.R’s role in the study prior to being approached. Seven recruiters agreed to take part. Six recruiters declined to take part or were unavailable. Seven semi-structured interviews were conducted by one interviewer (P.R.) by telephone between November 2022 and March 2023. The seven participants were a mixture of university (four) and NHS (three) employees. Confidentiality and anonymising of transcripts were emphasised to participants. The interviews took up to 80 min and were audio recorded using a Phillips digital pocket memo recorder and transcribed verbatim using the UK Transcription Service. Field notes were made. Transcripts were anonymised. Checking of transcripts by participants was not offered due to practical difficulties (staff turnover and staff constraints). Data were stored on a secure university drive.

The interviews explored the recruiters’ role in FEMuR III, including whether they were employed by the university or hospital, as well as the general practicalities around study recruitment. Topics discussed included barriers and facilitators to recruitment of patients and carers, satisfaction with the recruiter role, the impact of the COVID-19 pandemic upon recruitment and advice for future trials or suggestions for improving recruitment.

The analysis was undertaken using NVIVO software (Version 12) [11] by K.H. with a critical realist perspective used for the thematic analysis, considering the context and subjective experiences of participants [12]. One researcher (K.H.) coded the interview transcripts and grouped these into themes derived from the data with assistance from P.R. [12]. This involved immersion in the data systematically working through the transcripts to identify text which was relevant to the aim of the study, grouping the data into codes and developing themes. Reflection was used throughout by the interviewer and during analysis. K.H. also drew upon negative cases where individual interviewees disagreed with the consensus. A COREX checklist is available as an additional file (Additional File 1).

## Results

Six broad themes for both barriers and facilitators were identified: impact of COVID-19, access and integration, rapport and relationships, information and knowledge, perceptions of research and eligibility ambiguity (Table 1).

**Table 1:** Barrier and facilitators to recruitment into the FEMuR III randomised controlled trial

Themes	Barrier	Facilitator
Impact of COVID-19	- Access to carers - Access to patients/wards - Fear or reluctance	- Contact with others
Access and integration	- Access to wards/patients - Access to carers - Access to clinical notes - Ward turnover - Disconnect from main site - Time taken to complete recruitment tasks - Lack of capacity to deliver intervention - Differing priorities of staff- clinical versus research	- Access to carers - Clinical role - Access to clinical systems - Amendment to protocol to extend period of recruitment - Community recruitment - Persistence
Rapport and relationships	- Family influence	- Relationships with other clinical or administrative staff - Approaching with other staff (research or clinical) - Family Influence - Familiarity and rapport - Stress reduction
Information and knowledge	- Information overload - Lack of planning and guidance	- Training material
Perceptions of research	- Negative perception of research - Reluctance to take part - No carer identified/not resonating with label of carer	- Research knowledge
Eligibility ambiguity	- Difficulty separating clinical from research role - Concerns around capacity - Eligibility dilemmas	- Clear boundaries in clinical versus research role

### Impact of COVID-19

COVID-19 had a significant impact on recruitment with trial recruitment being halted on 19th March 2020, when only 96 participants had been recruited (21.5% of target sample), and recruitment remained variable depending on the region (due to local lockdowns) until the second quarter of 2022 when the trial ended. Even outside of these timeframes there were restrictions on non-essential staff entering the ward which limited recruiters’ opportunity to recruit. Resource priorities were transferred to COVID-19-related research during the pandemic which reduced the trial’s ability to meet recruitment targets.

Furthermore, carers and family members had restricted visiting to wards’ which made it difficult to recruit those individuals or for patients to discuss the trial with their family. When recruiters were able to approach patients, some patients were reluctant to have extra therapy sessions or have healthcare professionals in their home. A key factor in this decision-making appeared to be the fear of contracting COVID-19:

‘COVID was already something extra to deal with. They were already really stressed. The idea of having someone else come in or something else to deal with, people just said, “No. I’m not doing that.”’ (Participant 3).

However, three recruiters highlighted that the trial content motivated patients to take part as it included increased contact with others regardless of the trial arm they were allocated to. This may have been an encouraging effect during the isolation of the pandemic.

The remaining themes will focus on recruitment issues aside from the impact of COVID-19.

### Access and integration

Access and integration were recurring themes. This included access to clinical notes and systems, access to patients and their carers, differing priorities of staff (clinical versus research), disconnect between research sites and teams and time taken to complete research tasks.

Once potentially eligible patients were identified, the recruiter needed to review clinical notes to determine whether they met the eligibility criteria. The majority of recruiters reported practical difficulties in getting hold of this information, which was partly due to the variable access given to recruiters to clinical systems and notes. Once the patient was deemed to be eligible, approaching them also had its challenges due to the ward environment*:*

‘Mornings were the best times to get the handover sheets but weren’t necessarily the best times to recruit because obviously the patients were busy in the morning with the ward rounds and washing and dressing and whatever’ (Participant 3).

Similarly, access to carers on the ward could be difficult due to visiting restrictions. Patient turnover rates also influenced recruitment as patients could have moved wards or gone home between initial contact and subsequent approach for consent. However, a low turnover of patients reduced the number of potentially eligible patients being admitted. There was difficulty in following up patients if they moved ward during their stay due to recruiters having variable clinical system access, as demonstrated in the quote below:

‘Then when they went home we would keep tabs on their inpatient stay, which became a lot more difficult if they moved wards before they went home, because I didn’t have access to the hospital systems, because I’m only a researcher’ (Participant 3).

From a staff perspective, the recruitment process was labour and time intensive involving several visits to the ward and duplicate paperwork which was not captured in the screening logs. There were competing priorities depending on staffing role: clinical staff were busy and often unable to assist with recruitment due to ward pressures. One recruiter suggested that ward staff were best placed to recruit potential participants due to their familiarity with patients. However, due to time pressures, when they were asked to assist with recruitment eligible patients could be missed:

‘So, we tried that with a couple of the ward physios who were working, like I say, during the COVID bit, and actually we found that invariably they didn't have the time to do it’ (Participant 7).

In addition, feelings of being isolated from the main trial site were mentioned in several interviews. In addition, two recruiters described disconnect and communication difficulties between acute site recruitment and delivery of the intervention by community sites or healthcare partners and this was made more challenging by a lack of capacity to deliver the intervention in one site. It was suggested that meetings between sites specifically for recruiters, as happened between therapists, would have been helpful to develop ideas and learn from each other:

‘Just having a meeting, maybe once a week, just shows- Just strictly research staff that are dealing with recruitment, just to bounce ideas off each other and find out what’s working there and what isn’t working so well in other places’ (Participant 1).

Facilitators of recruiter access and integration included their having access to wards and clinical systems, having a clinical role, persistence and an amendment that extended the recruitment criteria. Access to clinical systems helped research staff to identify patients, sometimes remotely, which avoided repeated trips to the ward. Unsurprisingly, recruiters who were able to be on the ward at the same time as carers who were visiting had the most success in recruitment of carers, with recruiters also finding the presence of carers could be helpful for patient recruitment. Persistence was mentioned as a key factor in keeping recruitment momentum. In terms of the study itself, an amendment to extend the recruitment window (since time of surgery) from 4 to 6 weeks helped staff in their recruitment role. This change was made and approved on 03 February 2020 by the REC due to some potential participants being out of the window of recruitment before they were able to contemplate trial recruitment. This did not change the parameters of the RCT, but allowed some patient-participants to be recruited, who would otherwise be excluded.

### Rapport and relationships

All recruiters felt that the communication style with patients was key, focusing on rapport, body language and communication skills to explain the trial to the patient. Approaching patients with other healthcare professionals or research team members, particularly if the patient was already familiar with them, allowed for a relaxed conversation and the development of rapport:

‘Another thing is face to- I’ve put it here in the document, a face to a name. So, like I said, when you’re not just approaching them in one go, giving them all the information, you actually sit down and give your name and talk to them and approach them three or four times for little chats. I think that also helped improve because I think… I don’t know whether it’s the right phrase but I think they trusted you more’ (Participant 1).

Two of the seven recruiters were also involved in the intervention which may have impacted upon their recruitment style. One recruiter mentioned that giving a named contact of who would be visiting the patient appeared to help increase patient enthusiasm to take part.

Reducing stress for patients was seen as critical and this was done by reducing initial information (discussion with the patient and giving them the patient information leaflet before leaving them to read this) or recruiting in the home setting:

‘That worked better because they were home. They felt more comfortable. They were able to actually concentrate on what you were saying. They were really able to open up and ask questions, without beeping, and people coming in, left, right, and centre. So, it did feel like they had a better chance of informed consent when they were in their own home’ (Participant 7).

One recruiter pointed out that participants often only realised the limitations in their activities of daily living (ADLs) once they got home which meant that they were more receptive to the trial at this point. Recruiters also felt that being in a clinical role with clear boundaries helped them develop a relationship with potential participants. Developing relationships with clinical and clerical ward staff were key to helping them recruit:

‘Definitely having the relationship with the staff on the wards and getting to know some of the ward staff, like the therapists as well, because if you were querying, you know, cognition and things like that you could have a chat with them. Sometimes you would see things written in the notes and you were like, “Where is this person going afterwards?” We could have those conversations’ (Participant 3).

Patients would often discuss the trial with their family and their involvement could help or hinder recruitment, as summarised in the quote below:

‘It went one of two ways. It either meant they were encouraged, “Yes, you know, you should do it. Yes, (name), get signed up. I think it could be helpful,” or suspicious or “We don’t want you to do this. You’re going to have too much on.”’ (Participant 2).

### Information and knowledge

Knowledge and information about the trial among the patients, carers and recruiting staff was a key theme. Three staff discussed an initial lack of guidance around the recruitment process which meant that different sites implemented the trial differently (e.g. usual care was run alongside or prior to the intervention depending on site). If there were questions around eligibility, recruiters were not always hopeful of a quick response from the main site. The patient information sheet was mentioned several times as an example of issues not being picked up earlier in the trial:

‘(They) mentioned the information sheet, to begin with, having too much information on it and (they) also, I think, produced a document quite similar to mine, a simplified version, but that never got through to fruition for some reason I don’t understand. There were certain things like that,that I couldn’t believe weren’t picked up in the feasibility trial’ (Participant 1).

Six of the seven recruiters felt that providing patients with too much information initially could lead to them feeling overwhelmed. Patients eligible for recruitment had undergone major surgery and often had other stressors, including worries about the future and pain, which may have impacted upon their ability to take on new information about the trial, as demonstrated by the quote below:

‘Why don't you have a little read through that, and we'll come back to you in 2 days?” and they’re like, “I really feel rough. I'm on all kinds of medication for pain. I've just had an operation. What the hell are you asking me to read 18 pages for?”’ (Participant 7).

Using a shorter participation sheet was a recurring theme:

‘It just needs to be a larger font, and a very, “This is what I’m going to need you to do, if you’re in this trial, and this is what we don’t need from you.”’ (Participant 4).

Additionally, one recruiter felt that some questionnaires covered overlapping concepts and streamlining these could reduce questionnaire burden whilst still covering key concepts.

On a positive note, two recruiters commented that they regularly referred to the training and information material from the main site and found it helpful to prepare for recruiting.

### Perceptions of research

Patients could be reluctant to take part in the trial due to having a negative perception of research, for example one recruiter made a general observation that patients could perceive research as being treated as ‘lab rats’. Three out of seven recruiters mentioned that some potential participants did not understand about randomisation and wanted to be guaranteed receipt of usual care or the intervention. When it was explained to them that this was not possible, they did not want to take part in the trial:

‘Obviously, some of the barriers for them were if they were randomised, they may or may not get the intervention. Well, that’s just the study, isn’t it? You can’t change that’ (Participant 5).

Patients who had more knowledge of research, usually through previous experience or knowing a healthcare worker, were more likely to be interested in the trial:

‘What we found was a lot of patients that we did recruit, I think there were about six or seven that we recruited, had participated in research before. A few of them worked from universities in their life, teachers, so they were more than happy to participate’ (Participant 1).

One recruiter emphasised the ability to explain equipoise to a non-research audience*:*

‘You need to make sure that when you see these patients that you reiterate to them, “Actually, we don't know what the answer is. We don't know,” and that standard of care is just as important as an intervention, and why. That they're not actually going to lose anything out by taking part. They're still going to get their standard treatment’ (Participant 6).

Where present (i.e. not all patients have family), carers could also be reluctant to take part. For some this appeared to be due to their being unable to see a personal benefit or due to a reluctance to fill out questionnaires, but another reason suggested by one recruiter for carer non-recruitment was felt to be due to their not identifying as carers, i.e. the relative did not identify themselves in a caring role:

‘They’re (sic) very much saw a carer as somebody who was a paid carer that would come in to help mum get dressed or whatever, so on reading the information sheet, they would question, like, “I don’t think this is me.” I would, obviously, try and explain that they were the right type of person, but that definitely played into it, the label of ‘carer’’ (Participant 2).

It potentially shows a mismatch between medical/research language and the general population’s perception of the word ‘carer’. One recruiter added:

‘I didn’t recruit anyone into the carer side of things, mainly because I didn’t know much about it. It wasn’t really spoken about, I wasn’t sure what we were asking’ (Participant 4).

This highlights that recruiters may not have been clear on the inclusion criteria for carers.

### Eligibility ambiguity

Over half of recruiters reported ethical and moral dilemmas when deciding whether to approach patients, including concerns about patient capacity or patients who had other major life stresses, health problems, or expected reduced life expectancy. In such cases, recruiters may err on the side of caution and not approach the patient:

‘So if they had a cancer diagnosis, or they had quite severe heart failure, things that would indicate that they were probably within the last year of life, we would make a bit of a judgement call. We’d go, “Yes, we could recruit them, on paper they’re eligible, but is this a wise or kind thing to do?” to go in, knowing full well that…year’s follow-up for the trial, and they probably don’t want that burden in their lives’ (Participant 4).

However, one recruiter felt strongly that they should approach all eligible patients and give them the option to take part (according to the principles of an RCT):

‘There are people that I would go and actually take consent from, and I’d think, “They’re going to struggle with them in the community.” But you have to give the people a chance, don’t you? And if they fit all of the eligibility criteria, and they want to take part then they can take part’ (Participant 6).

Two recruiters mentioned difficulty in separating their clinical and research roles. Two recruiters mentioned that patients misunderstood their role and wanted clinical/therapeutic input from them. Research nurses felt that they could help clinically but were unable to in their recruitment role:

‘It’s just expectation management,and knowing that you probably could do something as a clinician there, but you can’t do something, because that’s not your job’ (Participant 4).

## Discussion

### Summary of main findings

The main barriers to recruitment for the FEMuR III trial were the lockdown restrictions imposed during the COVID-19 pandemic. However, 96/205 participants (46% of all recruited participants) were recruited pre-COVID so other recruitment issues have been explored. Recruitment staff identified the following barriers to recruitment: staffing difficulties, differing priorities of clinical staff, strong patient preference for or against the intervention, discussion around patient information leaflets, concerns about patient eligibility and equipoise and family members having difficulty identifying with the label of ‘carer’.

### Strengths and limitations

This paper described the challenges of recruiting into a multi-site RCT which ran in part during the COVID-19 pandemic time period. There were challenges due to COVID-19 lockdowns, limited access to wards to recruit both patients and carers and research focus shifting to COVID-19 studies. A variety of different staff involved in recruitment who worked as part of clinical and research teams were interviewed. However, this paper only considers the perspectives of recruiters who agreed to take part in these process evaluation interviews and not the perspectives of patients, carers, or other staff (e.g. clinical or ward staff). Furthermore, the views of the small sample of recruiters being interviewed may not have been representative of all the recruiters’ views. The interviews were also conducted post-recruitment which means that there was potential for recall bias. Transcript checking by participants was also not offered due to staff turnover and constraints.

### Comparison with previous literature

Prior to the COVID pandemic, recruitment to many RCTs of rehabilitation interventions has been challenging [8]. One systematic review concluded that recruitment in the community, following hospital discharge, was more likely to be successful after the immediate physical and psychological effects of their illness had abated [8]. COVID-19, however, brought additional challenges and recruitment to many other RCTs was reported to have been adversely affected by the pandemic with Lorenc et al. (2023) reporting that 11 out of 13 trials had lower recruitment rates than expected due to COVID-19 [13]. There were definitely challenges to recruitment from COVID in FEMuR III but 96/205 (46%) of the participants were recruited prior to COVID. A systematic review undertaken in 2014 which looked at the challenges of recruiting elderly and frail participants had some similar findings to this study including difficulty reading study and consent information, work pressures of recruitment staff and patient’s fear of unfamiliar people [14].

A minority of recruiters in the current study described ethical dilemmas in deciding who to approach to consent which could have resulted in selection bias. Fletcher et al. highlighted the paternalistic nature of this selective approach, which did not involve the patient in the decision-making process [15]. In a study (pre-COVID) which interviewed 72 healthcare professionals and chief investigators involved in six RCTs in a variety of different medical specialities, researchers found that recruiters were seemingly unaware of the negative impact on recruitment which stemmed from their own uncertainty around equipoise, uncertainty around patient eligibility, and conflict between their research and clinical roles [16]. Some recruiters in our study suggested that time would be better spent approaching only those patients who they felt were more likely to agree to take part (Table 2). This suggested a lack of understanding of the fundamental principles of an RCT and an unmet need for further training. The practicalities of conducting an RCT were covered in FEMuR III training for recruiters but not the underlying RCT principles. The recruiters also mentioned a disconnect between sites and felt that meetings would have been helpful. There were monthly drop-in sessions from June 2021 which were open to all site team members for discussion; however, these were stopped in February 2022 due to poor attendance. Refresher sessions for sites also took place after sites re-opened post-pandemic. Further training in RCT methodology, explanation of eligibility criteria and supporting staff to approach all eligible patients is needed in future rehabilitation trials [16].

**Table 2:** Suggestions from recruiters to improve future RCT recruitment

Topic	Practical Advice
Communication with patients	Explain equipoise clearly Reduce written information Reduce questionnaire burden/duplication Approach patients who are more likely to agree to take part
Guidance and Support	Recruitment guide Shorter/clearer Patient Information Leaflets
Reducing practical difficulties	Site-specific training Change window of recruitment to a later period after surgery Consider trial design e.g. Cluster RCT
Staff recruitment	Increased involvement of clinical staff in recruitment
Teamwork and Discussion	Meetings between recruiters at different sites
Time planning	Amending screening logs to account for time taken on recruitment tasks

Recruiters also noted that some patients had a strong preference about which treatment they were allocated (usual care versus intervention), a finding replicated by Donovan et al. (2016) [16]. This trial was not blinded for patients due to the nature of the intervention. One alternative trial design is patient preference, where those with a strong preference are allocated to that group and the remaining participants randomised. However, this does not appear to make a significant difference to recruitment [17, 18]. As suggested by one recruiter, a cluster randomised controlled trial may have been helpful in this situation and would help reduce the crossover between usual care and the intervention in some sites. One trial compared cluster randomisation with individual randomisation and found that cluster randomisation helped to improve recruitment [19]. This design would have allowed intervention resources to be focused in some centres and may have simplified recruitment, as the decision for intervention is made at a cluster level rather than individually. Equally this has its own challenges including reduced power which requires a larger sample size and knowledge of treatment group allocation prior to recruitment [19].

Carer recruitment was limited in the main trial with only 15 carers recruited as opposed to the feasibility study which recruited 31 carer-participants over a 10-month period from two sites [10]. It is likely that COVID was a major factor in carer recruitment issues with reduced access to wards for both potential carer participants and recruiters. However, there was a period of 11 months prior to the pandemic where carer recruitment has not replicated the rates seen in the feasibility study, despite the trial operating over multiple sites (as opposed to the initial two from the feasibility study).

Another possible explanation, highlighted by one recruiter, is that family members did not seem to identify with the label of ‘carer’ and this may have made them more reluctant to take part in the study. Studies have previously explored how the identity of ‘carer’ is formed [20–22]. Dobrof and Ebenstein found that carers often see personal care as part of a carer’s role but may not perceive other tasks such as providing support as ‘caring’ [22], being instead more part of a loving and reciprocal relationship between partners, or parent–child [23]. They may also view a carer as a purely professional role [24]. Hughes et al. conducted interviews with 40 family members or friends of people with multiple sclerosis (MS) to discuss their identity and feelings around the label of ‘carer’ [25]. Participants had a variety of responses to the label ‘carer’ with some embracing this term, others accepting it, some feeling that their roles changed depending on the care needs of the person with MS and some firmly rejecting the term. This can make it difficult to identify carers in studies. Finally, the trial protocol states that carers were defined as helping the patient for 4 or more days a week with activities of daily living or physical care [9]. This is a strict definition for carers and was not clearly highlighted in the carer information leaflet. The interviews suggest that recruiters may not have been clear on this definition themselves. The restricted definition could have made recruitment more challenging as it implies that carers needed to be involved consistently pre-admission.

For future trials looking at older patients with potential care needs, it is worth considering whether alternative terms are needed in order to resonate more with our target population. Broadening the definition of carer used in the trial could help to increase recruitment of potential carers, for example, to include those who provide ‘care provision…above and beyond that what is typical within the particular relationship’ [26]. Another option could be using an alternative term such as caregiver or family caregiver. Alternatively, interviewing family members to clarify their role in relation to the patient, but without using a label, may increase their recruitment and involvement in the trial thereby ensuring that the full spectrum of ‘carers’ perspectives are captured. Although the pandemic was a key barrier to carer recruitment, recruitment of carers was an issue in the 11-month period of recruitment prior to the pandemic and these are possible solutions for future complex rehabilitation trials.

A recurrent theme centred around reducing written information for patients and carers to read. This was implemented after a substantial amendment with REC and HRA approval in June 2020 with an 8-page patient information leaflet and a 6-page carer information leaflet (including consent forms). Ethical approval requires that sufficient information is provided for patients to give fully informed consent. Strategies to help with this, often suggested by recruiters, included several visits to the patients and introducing the trial verbally then leaving written information with the patient. Recruiters felt that the time spent recruiting was not always acknowledged and a log of how long it takes to recruit each patient would be helpful to allow for future recruitment planning in these complex rehabilitation trials (e.g. how many staff are needed).

Staffing was an issue during the study. This was felt particularly during the pandemic where there were staff shortages in many areas due to sickness or isolation rules and research staff being moved to COVID-19 studies. Interviewees mentioned general staff shortages in the hospitals as well as sickness and maternity leave as potential causes of staff fluctuation. Clinical staff were involved in recruitment but had to prioritise clinical care due to ward pressures, meaning that they were not always recruiting to their full potential. One site achieved effective recruitment but the community team (who were delivering the intervention) did not have the capacity to implement the intervention so recruitment was halted. Recruiters felt that involving the clinical team was important to increase rates of recruitment but acknowledged that this may be difficult to implement in practice, given that clinical teams do not often have protected research time. However, this challenge may be mitigated by having split clinical/research posts to ensure clinicians and nursing staff are embedded into research teams. This issue was evidenced in the systematic review by Fletcher et al. [15] which found that increased training and protected time of healthcare staff increased recruitment.

Ultimately, it is important to identify any recruitment issues early on during the trial. Embedding a qualitative study was found to be most effective for improving recruitment in one systematic review [15]. QRI-2 (QuinteT Recruitment Intervention) is a method for evaluating an RCT whilst in progress [27]. It involves two phases: Phase 1 evaluates the recruitment contemporaneously through documentation and interviews with patients and staff [28]. This identifies ‘clear obstacles’ and ‘hidden difficulties’ [28]. Phase 2 addresses recruitment difficulties by working with the key stakeholders including the trial management group to formulate a plan to improve recruitment [28]. Application of the QRI-2 method to 14 RCTs identified between three and six previously unrecognised issues per RCT [27]. Embedding qualitative research into the trial, with ongoing thematic analysis during the course of the trial to identify barriers and facilitators to recruitment, has also been demonstrated to be effective in improving recruitment rates [15, 29]. This could not have foreseen the COVID-19 pandemic but may help to identify issues early on in future trials on similar populations.

Table 2 groups the suggestions made by recruiters to improve recruitment in future RCTs. Suggestions included better communication with patients, better recruiter guidance and support, reduced practical difficulties, increased clinical staff recruitment, teamwork and discussion and time planning. However, not all suggestions are feasible and may go against the principles of an RCT.

### Implications for recruitment to future randomised controlled trials

This methodological paper analysed barriers and facilitators of recruitment to FEMuR III with six broad themes for both barriers and facilitators identified. COVID-19 was the most important factor in recruitment difficulties and this has been acknowledged. However, there were other barriers and facilitators to recruitment identified which were applicable to other trials of complex rehabilitation interventions. Strategies to help overcome these have been highlighted. These include alternative trial design (e.g. cluster RCT), broadening the definition of carer (or use of alternative terms), considering split clinical/research posts and acknowledging the time taken to recruit patients, particularly an older vulnerable populations who may need more assistance in understanding the trial or time to discuss with relatives. Our finding of the reluctance to approach people with comorbidities or complex social situations has important implications for recruiting vulnerable populations to similar trials and needs to be adequately addressed during training. It is key to identify and address issues early in the trial and this can be done through an embedded qualitative analysis, although this would not have foreseen the COVID-19 pandemic which was the main barrier to recruitment in the FEMuR III RCT. Suggestions by recruiters were considered, but some went against the principles of RCT, or were not feasible. It is important to consider the pressures on the health service and clinical staff which may limit the feasibility of split roles and availability of increased staff. These findings will help inform recruitment strategies in RCTs of complex rehabilitation in the future.

## Supplementary Information

Additional File 1. COREX checklist1.pdf.

## Data Availability

The datasets used and/or analysed during the current study are available from the corresponding author on reasonable request.

## Abbreviations

RCT: Randomised controlled trial
FEMuR III: Fracture in the Elderly Multidisciplinary Rehabilitation- Phase 3
COVID-19: Coronavirus Disease 2019
REC: Research Ethics Committee
HRA: Health Research Authority
PI: Principal Investigator
MS: Multiple sclerosis
ADL: Activities of daily living
QRI: QuinteT Recruitment Intervention
